# Two‐Dimensional Electromagnetic Navigation‐Assisted Percutaneous Transforaminal Endoscopic Discectomy for Lumbar Disc Herniation: A Retrospective Comparative Study

**DOI:** 10.1111/os.70441

**Published:** 2026-10-30

**Authors:** Zhong Wei, Haoyu Cheng, Ren Gao, Xuepeng Wang, Kangheng Niu, Zhenzhen Tao, Hao An, Biwang Huang, Chengjie Xiong, Junhong Lian

**Affiliations:** ^1^ Hubei University of Medicine Shiyan Hubei China; ^2^ Department of Orthopaedics, General Hospital of Central Theater Command Hubei Provincial Clinical Research Center for Orthpaedics Trauma Treatment Wuhan Hubei China; ^3^ The First School of Clinical Medicine, Southern Medical University Guangzhou Guangdong China

**Keywords:** learning curve, lumbar disc herniation, percutaneous transforaminal endoscopic discectomy, radiation exposure, three‐dimensional electromagnetic navigation, two‐dimensional electromagnetic navigation

## Abstract

**Objective:**

To compare the clinical performance, safety, radiation exposure, and learning curve of two‐dimensional (2D) electromagnetic navigation‐assisted percutaneous transforaminal endoscopic discectomy (PTED) with those of three‐dimensional (3D) electromagnetic navigation‐assisted PTED in patients with lumbar disc herniation (LDH).

**Methods:**

This retrospective cohort study included 82 patients with LDH who underwent PTED between January 2022 and June 2024. Patients were classified into the 2D electromagnetic navigation group or the 3D electromagnetic navigation group according to the navigation workflow used during surgery. Perioperative variables, including total operative time, navigation registration time, radiation exposure, and complications, were recorded. Clinical outcomes were assessed using the visual analogue scale (VAS), Oswestry Disability Index (ODI), and modified MacNab criteria. Learning curves for both the surgeon and the radiologic technologist were evaluated using cumulative sum (CUSUM) analysis.

**Results:**

Compared with the 3D group, the 2D group demonstrated a significantly shorter total operative time (66.64 ± 9.67 vs. 88.92 ± 15.98 min, *p* < 0.001), shorter navigation registration time (6.37 ± 0.93 vs. 12.25 ± 2.11 min, *p* < 0.001), and lower radiation exposure (1.77 ± 0.17 vs. 7.24 ± 0.56 mGy, *p* < 0.001). Longitudinal analysis using linear mixed‐effects models demonstrated significant time effects for VAS of back, VAS of leg, and ODI (all *p* < 0.001), indicating significant postoperative improvement in both groups. No significant main effect of group was observed for VAS of back, VAS of leg, or ODI. The group‐by‐time interaction was not significant for VAS of leg (*p* = 0.774), but was significant for VAS of back (*p* = 0.007) and ODI (*p* < 0.001), suggesting differences in the temporal recovery patterns between groups for these two outcomes. According to the modified MacNab criteria, favorable final outcomes were achieved in both groups, with no significant between‐group difference. The learning curve was shorter in the 2D group, with proficiency reached by the 16th case for the surgeon and the 14th case for the radiologic technologist, compared with the 3D group. No significant between‐group difference was observed in complication rates.

**Conclusion:**

2D electromagnetic navigation‐assisted PTED is a safe and clinically effective alternative to 3D electromagnetic navigation‐assisted PTED for the treatment of LDH. Relative to the 3D workflow, the 2D approach offers greater procedural efficiency, reduced radiation exposure, and a shorter learning curve. Although longitudinal analysis revealed different recovery patterns over time for low back pain and ODI between the two groups, both techniques achieved substantial postoperative improvement, favorable final clinical outcomes, and comparable safety. These findings suggest that simplified 2D electromagnetic navigation may represent a more practical and scalable option for routine clinical application.

## Graphical abstract

This study compares 2D versus 3D electromagnetic navigation‐assisted PTED for lumbar disc herniation. The 2D system significantly reduced surgical time, radiation exposure, and learning curve while maintaining similar clinical outcomes. It offers an efficient, safe, and practical alternative for less experienced surgeons.

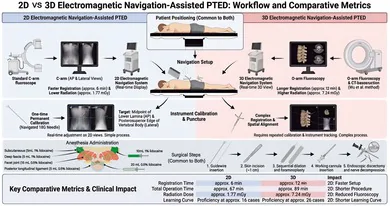

## 1 Introduction

Lumbar disc herniation (LDH) results in the compression of nerve roots, leading to conditions such as low back pain or sciatica, affecting approximately 600 million individuals globally [1, 2]. In the United States, the economic burden of low back pain annually ranges from billions to hundreds of billions of dollars [3]. Although conventional open surgery is effective in mitigating clinical symptoms, it is associated with notable complications, including infection and limitations in segmental motion [4, 5]. In recent years, percutaneous transforaminal endoscopic discectomy (PTED) has gained prominence and is widely recognized as a safe and effective technique [6, 7, 8, 9]. This approach significantly minimizes surgery‐related trauma, thereby reducing postoperative pain and enhancing functional recovery [10, 11]. However, conventional PTED is regarded as a procedure heavily reliant on the surgeon's experience, characterized by a steep learning curve [12]. The necessity for repeated punctures under C‐arm guidance to ascertain the optimal positioning of the working cannula not only elevates radiation exposure for both medical personnel and patients but also increases the risk of nerve root injury [13, 14]. Therefore, acquiring fundamental technical competencies within a limited timeframe is the principal objective for spine surgeons.

It has been reported that navigation may alleviate challenges and expedite the learning process associated with PTED [15]. With advancements in spine navigation technology, various navigation systems have been developed to support PTED procedures [16]. Optical navigation represents the predominant navigation system in contemporary surgical practice, utilizing infrared light to track surgical instruments for precise positioning. Nevertheless, its effectiveness is compromised by the potential for physical obstruction of infrared signals [17]. In contrast, electromagnetic navigation employs electromagnetic field sensing to ascertain the needle tip position of puncture instruments; the needle may bend during the puncture process, making it particularly suitable for the puncture process in PTED surgery [18]. However, current mainstream surgical navigation systems heavily rely on preoperative and intraoperative three‐dimensional (3D) imaging [14, 19]. The processes involved in 3D image reconstruction and instrument registration substantially increase the complexity of surgical procedures and extend operation times, thereby impeding enhancements in surgical efficiency.

Although navigation‐assisted PTED has been increasingly applied in the treatment of lumbar disc herniation, the current literature has largely focused on comparisons between navigation‐guided procedures and conventional fluoroscopy‐guided techniques, or on technical descriptions of emerging navigation platforms. Previous studies have reported the use of electromagnetic navigation, real‐time 3D electromagnetic navigation, integrated optical‐magnetic navigation, and instant X‐ray–based navigation systems in endoscopic lumbar surgery. Nevertheless, direct comparative evidence between different electromagnetic navigation workflows remains limited, and to our knowledge based on the currently available literature, no clinical study has specifically compared two‐dimensional and three‐dimensional electromagnetic navigation in PTED for lumbar disc herniation. Building on our previous experience with 3D electromagnetic navigation‐assisted PTED, we further refined the workflow by implementing a simplified 2D electromagnetic navigation strategy based on intraoperative anteroposterior and lateral fluoroscopic image registration, followed by real‐time electromagnetic guidance throughout the puncture process (Figure 1). The present study was therefore conducted to compare 2D and 3D electromagnetic navigation‐assisted PTED, with particular attention to procedural efficiency, radiation exposure, learning curve, clinical outcomes, and safety. We hypothesized that 2D electromagnetic navigation would achieve favorable clinical outcomes and safety while offering advantages in workflow simplification and operative efficiency.

**FIGURE 1:**
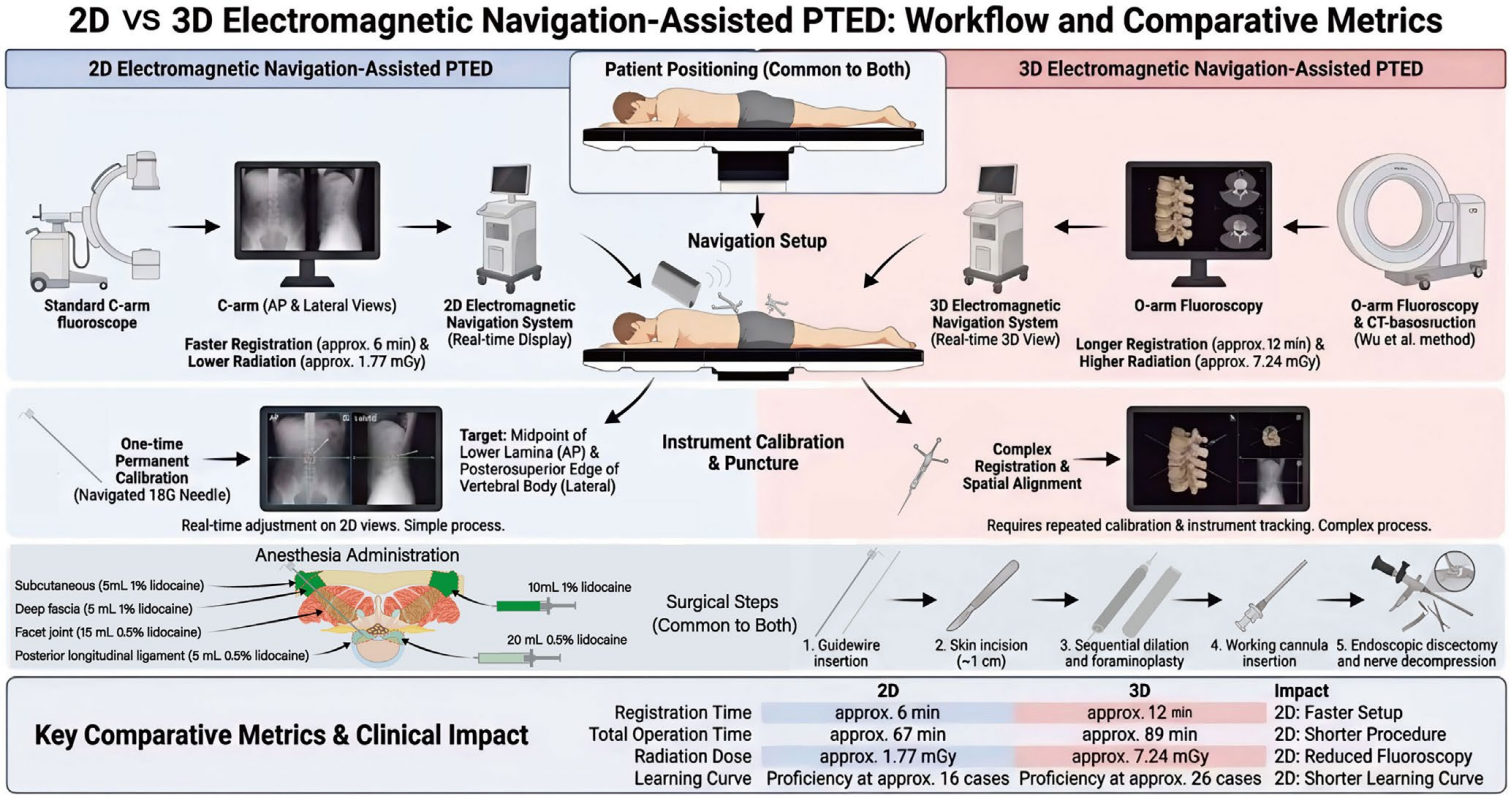
Comparison of 2D and 3D electromagnetic navigation‐assisted PTED: Schematic overview.

## 2 Methods

### 2.1 Inclusion and Exclusion Criteria

Inclusion Criteria: (1) Age between 18 and 65 years; (2) Single‐level LDH with associated foraminal stenosis and/or lateral recess stenosis; (3) Clinical symptoms consistent with imaging findings; (4) Failure of at least 3 months of conservative treatment; (5) Available follow‐up data for at least 12 months postoperatively, with complete medical records. Exclusion Criteria: (1) Spinal instability, tuberculosis, or severe osteoporosis; (2) History of malignancy, systemic infections, or coagulation/immunological disorders; (3) Significant disc calcification on imaging; (4) Previous lumbar spine surgery; (5) Pregnant or breastfeeding women.

### 2.2 Patient Population

This retrospective study included patients who underwent PTED between January 2022 and June 2024. The study was approved by the institutional ethics committee (Approval No. [2026]027‐01) and conducted in accordance with the Declaration of Helsinki. Written informed consent was obtained from all patients prior to enrollment. According to the inclusion and exclusion criteria, 82 eligible patients were enrolled. Patients were classified into the 2D navigation group or the 3D navigation group according to the navigation workflow used during surgery. Before surgery, all patients were informed of both treatment options, and the final treatment selection was made through shared decision‐making between the surgeon and the patient. All procedures included in this study were performed by the same senior spinal surgeon throughout the study period.

### 2.3 Surgical Technique

#### 2.3.1 2D Navigation Group

The patient was placed in a prone position on a carbon fiber table, which is resistant to electromagnetic interference. Soft pillows were positioned under the patient's shoulders and anterior superior iliac spines to maintain a neutral spinal alignment. After fixation of the reference frame, posteroanterior and lateral fluoroscopic images of the lumbar spine were obtained using a mobile C‐arm and transferred to the 2D electromagnetic navigation system. The magnetic field generator, connected to a free arm, was positioned on the contralateral side of the patient, directly facing the reference frame (Figure 2A).

**FIGURE 2:**
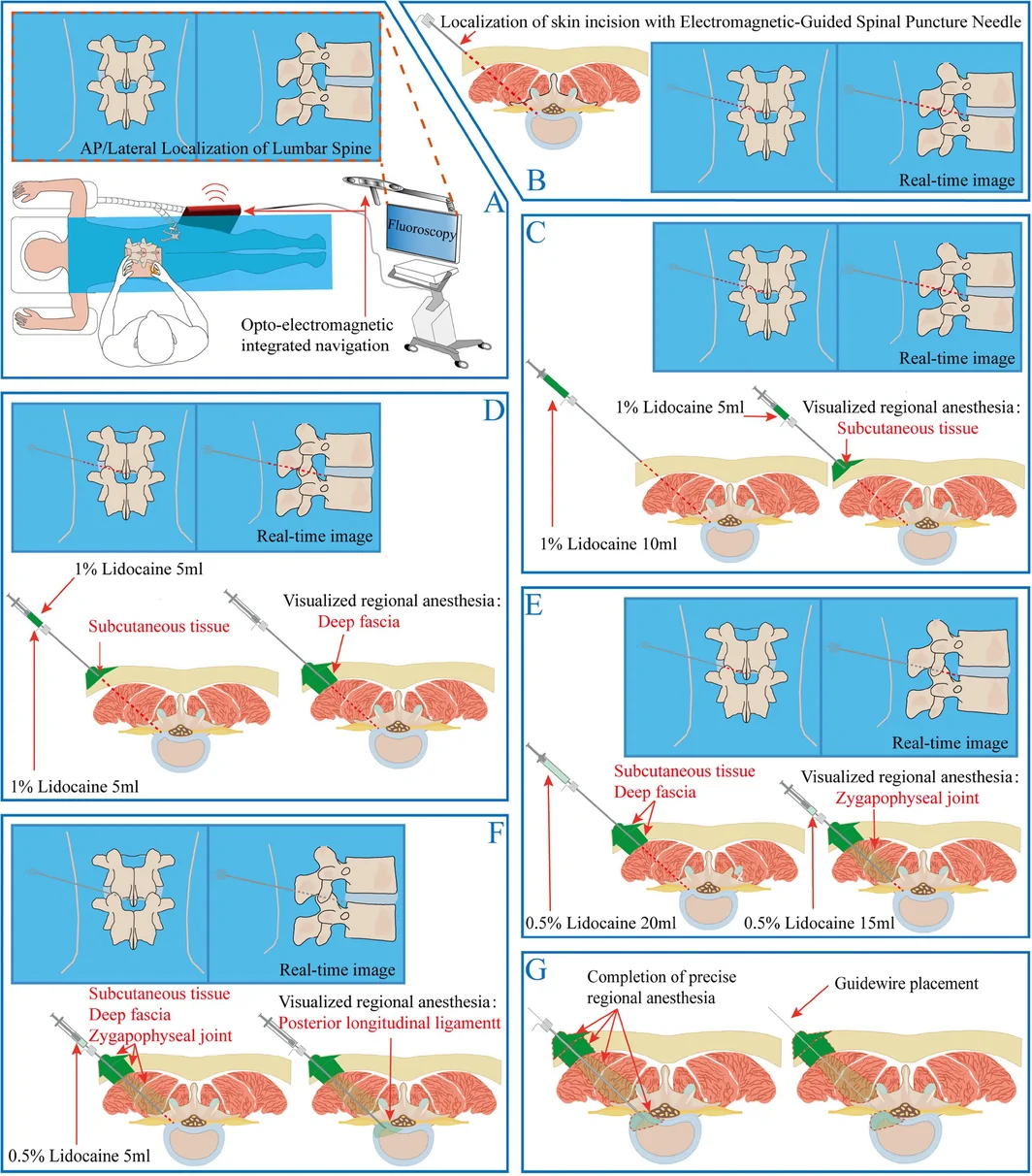
The intraoperative navigation registration and precise regional anesthesia schematic. (A) After fixation of the reference frame, the posteroanterior and lateral fluoroscopic images of the lumbar spine are transferred to the 2D electromagnetic navigation system. (B) Under real‐time imaging guidance, a needle is positioned at the skin incision site using the electromagnetic‐guided puncture needle. (C) Subcutaneous anesthesia is administered using 1% lidocaine (5 mL) under real‐time imaging guidance. (D) Deep fascial anesthesia is administered using 1% lidocaine (5 mL) under real‐time imaging guidance. (E) Zygapophyseal joint anesthesia is performed using 0.5% lidocaine (15 mL) under real‐time imaging guidance. (F) Posterior longitudinal ligament anesthesia is administered using 0.5% lidocaine (5 mL) under real‐time imaging guidance. (G) The navigation system provides real‐time imaging for the precise completion of anesthesia in each region, followed by the placement of the guidewire.

Once it was confirmed that the navigated 18G puncture needle could receive the magnetic field signal, the needle was calibrated, and the surgical procedure commenced. The skin incision was determined based on the navigation‐guided trajectory (Figure 2B). After marking the skin incision site, subcutaneous anesthesia was administered using 1% lidocaine (5 mL) under real‐time navigation guidance (Figure 2C). The navigated 18G puncture needle was then introduced through the skin. The needle trajectory was adjusted in real time according to its position on the navigated posteroanterior and lateral views. The target on the posteroanterior view was the midpoint of the lower lamina of the responsible level, and on the lateral view, it was the posterosuperior edge of the vertebral body at the responsible level. Appropriate adjustments to the puncture trajectory were made according to the location of the disc protrusion based on the MSU classification. During needle advancement, deep fascial anesthesia was intermittently administered using 1% lidocaine (5 mL) under real‐time navigation guidance (Figure 2D).

When the 2D electromagnetic navigation system indicated that the needle tip had reached the zygapophyseal joint, and after aspiration confirmed no blood return, 0.5% lidocaine (15 mL) was injected to perform zygapophyseal joint anesthesia under real‐time navigation guidance (Figure 2E). If no significant resistance was encountered, the 18G needle was advanced further until it reached the final target position, confirmed on the posteroanterior view as the midpoint of the lower lamina and on the lateral view as the posterosuperior edge of the vertebral body. At this point, posterior longitudinal ligament anesthesia was administered using 0.5% lidocaine (5 mL) under real‐time navigation guidance (Figure 2F). Intraoperative adjustments to the puncture trajectory were made as needed, considering the patient's body habitus (e.g., obesity) and the type of disc herniation.

After completion of navigation‐guided regional anesthesia, the stylet was removed from the 18G puncture needle (Figure 2G), and a guidewire was inserted through the needle. A guidewire was inserted through the needle, after which the needle was withdrawn. An approximately 1 cm skin incision was made at the guidewire entry point. Sequential dilators were used to dissect through the skin, fascia, and muscle layers. Corresponding reamers were used alongside the dilators to perform foraminoplasty, enlarging the intervertebral foramen to accommodate the working cannula. Following adequate foraminoplasty, the working cannula was inserted. Under the visualization of the spinal endoscope system, the herniated nucleus pulposus was removed. Partial resection of the ligamentum flavum was performed to achieve dorsal decompression. The nerve root was carefully explored both cranially and caudally to ensure complete decompression, confirmed by the restoration of visible pulsation synchronous with respiration. Potential bleeding points were meticulously identified and coagulated using a monopolar electrocautery device until no active bleeding was observed under endoscopy. Finally, the endoscopic working cannula and the endoscope system were withdrawn, and the skin incision was sutured.

#### 2.3.2 3D Navigation Group

We adopted the method described by Wu et al. [14], which utilized a real‐time 3D electromagnetic navigation system for puncture and instrumentation during PTED.

#### 2.3.3 Postoperative Management

Postoperative management was standardized for all patients. Analgesics and neurotrophic medications were administered to facilitate recovery. Patients were encouraged to perform straight leg raise exercises (10 repetitions/set, 3 sets/day) and lumbar and back muscle strengthening exercises (5 repetitions/set, 3 sets/day) progressively, increasing the intensity and frequency based on their recovery status. If patients did not report significant radicular pain, they were encouraged to ambulate with a lumbar brace protection starting from the second postoperative day, with daily activity limited to less than 30 min initially. The lumbar brace was typically worn for 1–2 months. Strenuous physical activities were avoided for the first month, while appropriate exercises such as swimming were encouraged as tolerated.

### 2.4 Clinical Assessment

We collected the demographic data of the patients, including age, sex, body mass index (BMI), duration of symptoms, American Society of Anesthesiologists (ASA) physical status classification, herniation type (MSU classification), side of disc herniation (left/right), herniated segment, and follow‐up duration. Perioperative evaluation parameters included total surgical time, navigation registration time, puncture and catheter placement times, intraoperative radiation dose, intraoperative pain, and postoperative length of hospital stay. Intraoperative pain was categorized as follows: “Mild” = mild or no pain at the puncture site, “Moderate” = moderate pain from the puncture lasting a short duration, “Severe” = intense pain from the puncture lasting a longer period, and “Intolerable” = pain so severe that the procedure should be stopped. Clinical assessments were conducted to evaluate patients' functional status preoperatively and at follow‐up visits (1 week, 3 months, and final follow‐up), using the visual analog scale (VAS) [20] and Oswestry Disability Index (ODI) [21]. VAS and ODI scores were recorded at each orthopedic outpatient visit. Overall satisfaction was assessed using the modified MacNab criteria [7, 22] (excellent, good, fair, poor). To accurately reflect postoperative pain and activity limitations, which could not be fully assessed during surgery, the modified MacNab criteria were re‐assessed at the final follow‐up (more than 1 year postoperatively). Recurrence of symptoms, complications, and other adverse events during the follow‐up period were also recorded. Clinical assessments were performed preoperatively and at 1 week, 3 months, and the final follow‐up visit. The final follow‐up was defined as the last available outpatient assessment conducted at least 12 months after surgery.

### 2.5 Learning Curve Analysis

To minimize operator‐related heterogeneity, all cases included in the surgeon learning‐curve analysis were performed by the same senior spinal surgeon throughout the study period. The cumulative sum (CUSUM) method was used to evaluate the learning curves for both the surgeon and the radiologic technologist [23]. CUSUM analysis was performed separately for the 2D navigation group and the 3D navigation group, with cases arranged in chronological order according to the sequence in which the procedures were performed.

For the surgeon, the performance indicator was defined as the total operative time for each case. For the radiologic technologist, the performance indicator was defined as the navigation registration time for each case. The CUSUM value for the i‐th case was calculated as follows [24]:

$$\text{CUSUM}=\sum_{i=1}^{n}\left(x_{i}-\mu \right)$$

where $x_{i}$ represents the observed time of the *i*‐th case and *μ* represents the mean time of all cases in the corresponding group. A positive increment indicated that the observed value was above the group mean, whereas a negative increment indicated that it was below the group mean.

CUSUM curves were plotted with case sequence on the *x*‐axis and CUSUM value on the *y*‐axis using Origin 2024 software. A fitted curve was then generated, and the peak point of the fitted CUSUM curve was defined as the turning point, indicating the transition from the learning phase to the proficiency phase. Cases before the turning point were considered to represent the learning phase, whereas cases after the turning point were considered to represent the proficiency phase. The goodness of fit was assessed using the coefficient of determination (*R* ^2^), with higher *R* ^2^ values indicating a better fit.

### 2.6 Statistical Analyses

Statistical analysis was performed using SPSS version 27.0 (SPSS Inc., Chicago, IL, USA). Continuous variables with a normal distribution were expressed as mean ± standard deviation, and categorical variables were presented as frequencies and percentages. Between‐group comparisons for baseline and perioperative continuous variables were performed using the independent‐samples *t*‐test, while categorical variables were analyzed using the chi‐square test or Fisher's exact test, as appropriate. When continuous variables did not satisfy the normality assumption, the Mann–Whitney *U* test was used.

For longitudinally measured continuous outcomes, including VAS of back, VAS of leg, and ODI, linear mixed‐effects models were used to account for within‐subject correlations across repeated assessments. Group, time, and the group‐by‐time interaction were included as fixed effects, and patient was treated as a random effect. Post hoc comparisons between preoperative and postoperative time points within each group were adjusted using the Bonferroni method. A two‐sided *p* value < 0.05 was considered statistically significant.

## 3 Results

### 3.1 Demographic Variables

A total of 82 patients were included in this study, all of whom met the inclusion and exclusion criteria. Among these patients, 35 underwent PTED assisted by a 2D electromagnetic navigation system, and 47 underwent PTED assisted by a 3D electromagnetic navigation system. The baseline characteristics of the two groups were comparable, with no significant differences (*p* > 0.05) (Table 1). In terms of herniation type, 40% of patients in the 2D navigation group had Type II herniation, while 60% had Type III herniation. In the 3D navigation group, 44.7% had Type II herniation and 55.3% had Type III herniation. With respect to the side of herniation, 54.3% of patients in the 2D navigation group had left‐sided herniations, and 45.7% had right‐sided herniations. In the 3D navigation group, 51.1% had left‐sided herniations, and 48.9% had right‐sided herniations. As for the herniated segments, 54.3% of patients in the 2D navigation group had herniation at the L4–L5 level, and 31.4% had it at the L5–S1 level. In the 3D navigation group, 46.8% had herniation at the L4–L5 level, and 38.3% at the L5–S1 level. The duration of follow‐up was also comparable between the two groups (13.39 ± 1.03 vs. 13.64 ± 0.82 months, *p* = 0.217).

**TABLE 1:** Comparison of baseline characteristics between the two groups.

Characteristic	2D Navigation group (*N* = 35)	3D Navigation group (*N* = 47)	*p*
Age (years)	43.37 ± 13.45	46.09 ± 13.67	0.373
Gender			0.824
Male	19 (54.3)	27 (57.4)	
Female	16 (45.7)	20 (42.6)	
BMI (kg/m^2^)	23.02 ± 3.40	24.14 ± 4.07	0.191
Duration of symptoms (months)	11.26 ± 5.42	10.40 ± 6.54	0.393
ASA physical status classification			0.623
ASA (I/II)	24 (68.6)	35 (74.5)	
ASA III	11 (31.4)	12 (25.5)	
Herniated type (MSU classification)			0.822
II	14 (40)	21 (44.7)	
III	21 (60)	26 (55.3)	
Side			0.656
Left	19 (54.3)	22 (46.8)	
Right	16 (45.7)	25 (53.2)	
Herniated segment			0.780
Other segments	5 (14.3)	7 (14.9)	
L4‐L5	19 (54.3)	22 (46.8)	
L5‐S1	11 (31.4)	18 (38.3)	
Duration of follow‐up (months)	13.39 ± 1.03	13.64 ± 0.82	0.217

*Note:* Values are presented as mean ± standard deviation or number (%).

### 3.2 Perioperative Indicators

A comparison of the perioperative data between the two groups showed significant differences across several parameters (Table 2). Compared to the 3D navigation group, the 2D navigation group had a significantly shorter total operative time (66.64 ± 9.67 min vs. 88.92 ± 15.98 min; *p* < 0.001). Similarly, the navigation registration time in the 2D navigation group was significantly shorter than that in the 3D navigation group (6.37 ± 0.93 min vs. 12.25 ± 2.11 min; *p* < 0.001). Additionally, the radiation dose in the 2D navigation group was also significantly lower than in the 3D navigation group (1.77 ± 0.17 mGy vs. 7.24 ± 0.56 mGy; *p* < 0.001). However, there were no significant differences between the two groups in terms of puncture and catheter placement time, intraoperative pain, or postoperative hospital stay (*p* > 0.05).

**TABLE 2:** Comparison of perioperative parameters between the two groups.

Characteristic	2D Navigation group (*N* = 35)	3D Navigation group (*N* = 47)	*p*
Total operation time (min)	66.64 ± 9.67	88.92 ± 15.98	< 0.001
Navigation registration time (min)	6.37 ± 0.93	12.25 ± 2.11	< 0.001
Puncture and catheter placement time (min)	19.46 ± 3.93	20.33 ± 5.02	0.377
Radiation dose (mGy)	1.77 ± 0.17	7.24 ± 0.56	< 0.001
Intraoperative pain			0.378
Mild	25 (71.4)	26 (55.3)	
Moderate	9 (25.7)	16 (34.0)	
Severe	1 (2.9)	4 (8.5)	
Intolerable	0	1 (2.1)	
Postoperative hospital stay (days)	2.86 ± 0.81	2.78 ± 0.77	0.609

*Note:* Values are presented as mean ± standard deviation or number (%).

### 3.3 Learning Curve Comparison Between Groups

CUSUM curves were constructed separately for the surgeon and the radiologic technologist in the 2D and 3D navigation groups according to chronological case order. The learning curves were represented by the best‐fitted third‐order polynomial curves, as shown in Figure 3A–D. In general, the initial upward phase of the CUSUM curve reflected operative or registration times that were relatively longer than the corresponding group mean, whereas the subsequent downward phase indicated progressive improvement in procedural efficiency.

**FIGURE 3:**
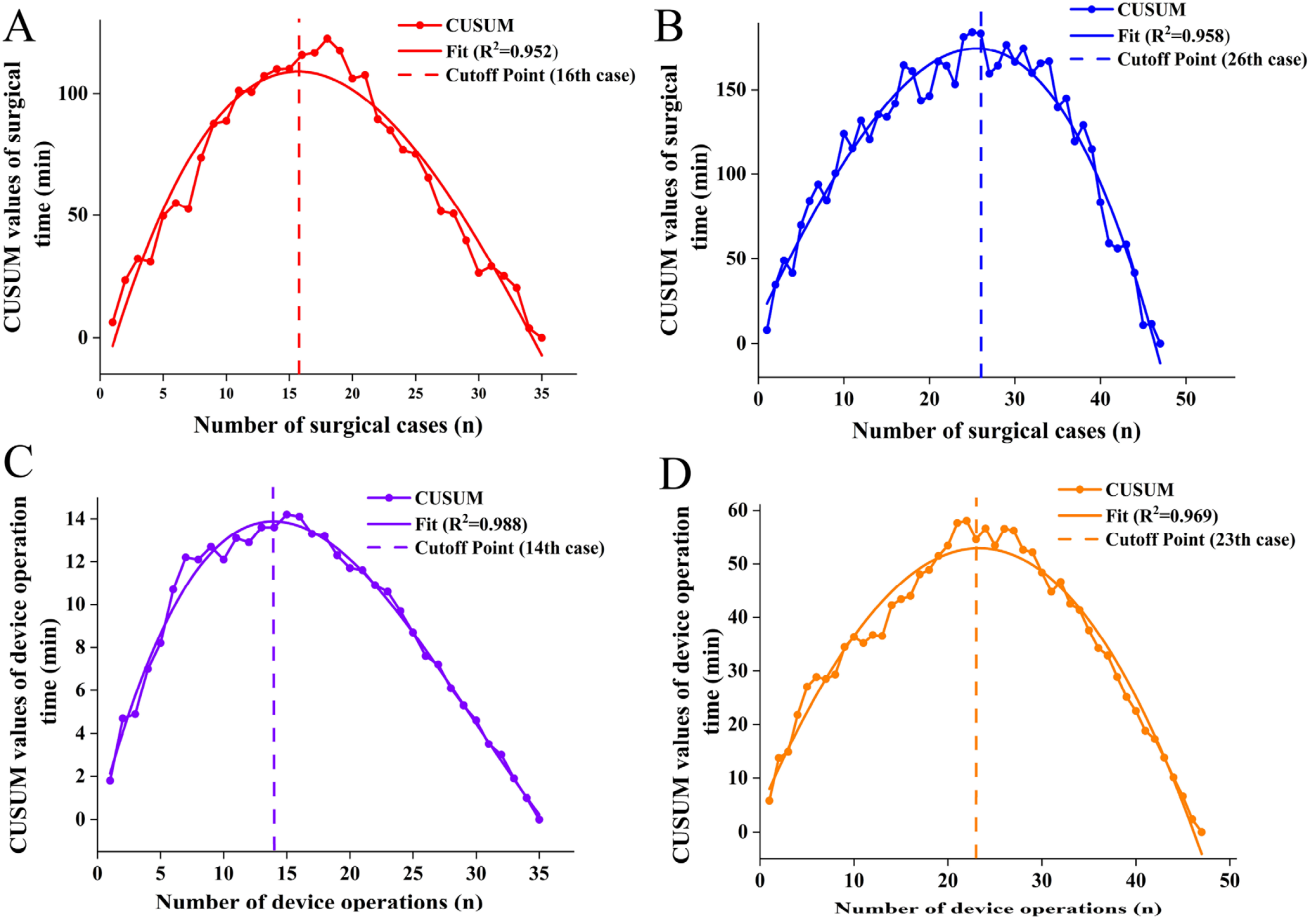
CUSUM learning curves of the surgeon and radiologic technologist. (A) CUSUM learning curve of the surgeon in the 2D navigation group, based on total operative time. (B) CUSUM learning curve of the surgeon in the 3D navigation group, based on total operative time. (C) CUSUM learning curve of the radiologic technologist in the 2D navigation group, based on navigation registration time. (D) CUSUM learning curve of the radiologic technologist in the 3D navigation group, based on navigation registration time. The peak of each fitted CUSUM curve was defined as the turning point between the learning phase and the proficiency phase.

For the surgeon, the turning points of the fitted CUSUM curves were identified at the 16th case in the 2D group (*R* ^2^ = 0.952; Figure 3A) and the 26th case in the 3D group (*R* ^2^ = 0.958; Figure 3B), suggesting that proficiency was achieved earlier with the 2D workflow. For the radiologic technologist, the turning points occurred at the 14th case in the 2D group (*R* ^2^ = 0.988; Figure 3C) and the 23rd case in the 3D group (*R* ^2^ = 0.969; Figure 3D), again indicating a shorter learning process in the 2D group.

These findings suggest that the 2D electromagnetic navigation workflow was associated with a shorter learning curve than the 3D workflow for both the surgeon and the radiologic technologist.

### 3.4 Clinical Outcomes

There were no significant differences in preoperative VAS of back, VAS of leg, or ODI scores between the two groups. Longitudinal analysis using linear mixed‐effects models demonstrated significant time effects for VAS of back, VAS of leg, and ODI (all *p* < 0.001), indicating significant postoperative improvement in both groups. No significant main effect of group was observed for VAS of back (*p* = 0.798), VAS of leg (*p* = 0.259), or ODI (*p* = 0.487).

With respect to the group‐by‐time interaction, no significant interaction was observed for VAS of leg (*p* = 0.774), suggesting similar temporal recovery patterns between the two groups for radicular pain. However, significant group‐by‐time interactions were identified for VAS of back (*p* = 0.007) and ODI (*p* < 0.001), indicating differences in the pattern of improvement over time between the two groups for these two outcomes. Nevertheless, post hoc comparisons showed that both groups achieved significant improvements in all postoperative VAS and ODI scores compared with the preoperative values after Bonferroni correction (all adjusted *p* < 0.001) (Table 3).

**TABLE 3:** Longitudinal analysis of VAS and ODI outcomes between the two groups.

Outcome	Time	2D Navigation group (*N* = 35)	3D Navigation group (*N* = 47)	P time	P group	P group × time
VAS of back	Preoperative	5.71 ± 1.05	5.42 ± 0.97	< 0.001	0.798	0.007
	1 week	2.49 ± 0.51	2.45 ± 0.58			
	3 months	1.46 ± 0.51	1.40 ± 0.54			
	Final follow‐up	1.14 ± 0.55	1.26 ± 0.57			
VAS of leg	Preoperative	6.17 ± 1.10	6.40 ± 1.12	< 0.001	0.259	0.774
	1 week	2.77 ± 0.73	2.98 ± 0.82			
	3 months	1.69 ± 0.63	1.79 ± 0.78			
	Final follow‐up	1.09 ± 0.56	1.23 ± 0.70			
ODI score	Preoperative	59.66 ± 6.50	61.15 ± 7.42	< 0.001	0.487	< 0.001
	1 week	26.34 ± 6.03	25.40 ± 7.11			
	3 months	13.94 ± 5.08	14.55 ± 5.62			
	Final follow‐up	11.26 ± 4.95	10.38 ± 5.30			

*Note:* Values are presented as mean ± standard deviation. Longitudinal changes in VAS of back, VAS of leg, and ODI were analyzed using linear mixed‐effects models, with group, time, and the group‐by‐time interaction entered as fixed effects and patient entered as a random effect. Post hoc comparisons between preoperative and postoperative time points within each group were adjusted using the Bonferroni method.

At the final follow‐up, the modified MacNab criteria showed favorable outcomes in both groups, with no significant difference between groups (*p* = 0.518). Complications including tear of the dural sac, sensory abnormalities in the lower limbs, and recurrence were observed in both groups, with no significant difference between groups (Table 4).

**TABLE 4:** Comparison of the modified MacNab criteria and complications between the two groups.

Characteristic	2D Navigation group (*N* = 35)	3D Navigation group (*N* = 47)	*p*
Modified MacNab			0.518
Excellent	31 (88.6)	36 (76.6)	
Good	3 (8.6)	8 (17.0)	
Fair	1 (2.9)	2 (4.3)	
Poor	0	1 (2.1)	
Complications			0.830
None	31 (88.6)	40 (85.1)	
Tear of the dural sac	1 (2.9)	2 (4.3)	
Sensory abnormalities in the lower limbs	3 (8.6)	4 (8.5)	
Recurrence	0	1 (2.1)	

*Note:* Values are presented as number (%).

### 3.5 Complications

Some minor complications were noted in the patients of both surgical groups. In the 2D navigation group, 1 patient (1/35) experienced a tear of approximately 3 mm in the dural membrane without cerebrospinal fluid leakage and recovered after bed rest. Additionally, 3 patients (3/35) reported transient lower limb numbness postoperatively, which resolved within 4 days. In the 3D navigation group, 2 patients (2/47) experienced a tear of approximately 2 mm in the dural membrane without cerebrospinal fluid leakage and recovered after bed rest. Four patients (4/47) reported postoperative lower limb numbness, which resolved within 1 week. Furthermore, 1 patient (1/47) experienced symptom recurrence 3 months after surgery and required an open fusion procedure.

## 4 Case Report

A 38‐year‐old female patient presented with left lower back pain persisting for 2 years, accompanied by pain and numbness along the left lateral calf and dorsum of the foot. Prior to admission, the patient had undergone conventional conservative treatment, but symptoms recurred. Preoperative X‐ray, CT, and MRI scans revealed disc herniation at the L5–S1 level (MSU III) (Figure 4A).

**FIGURE 4:**
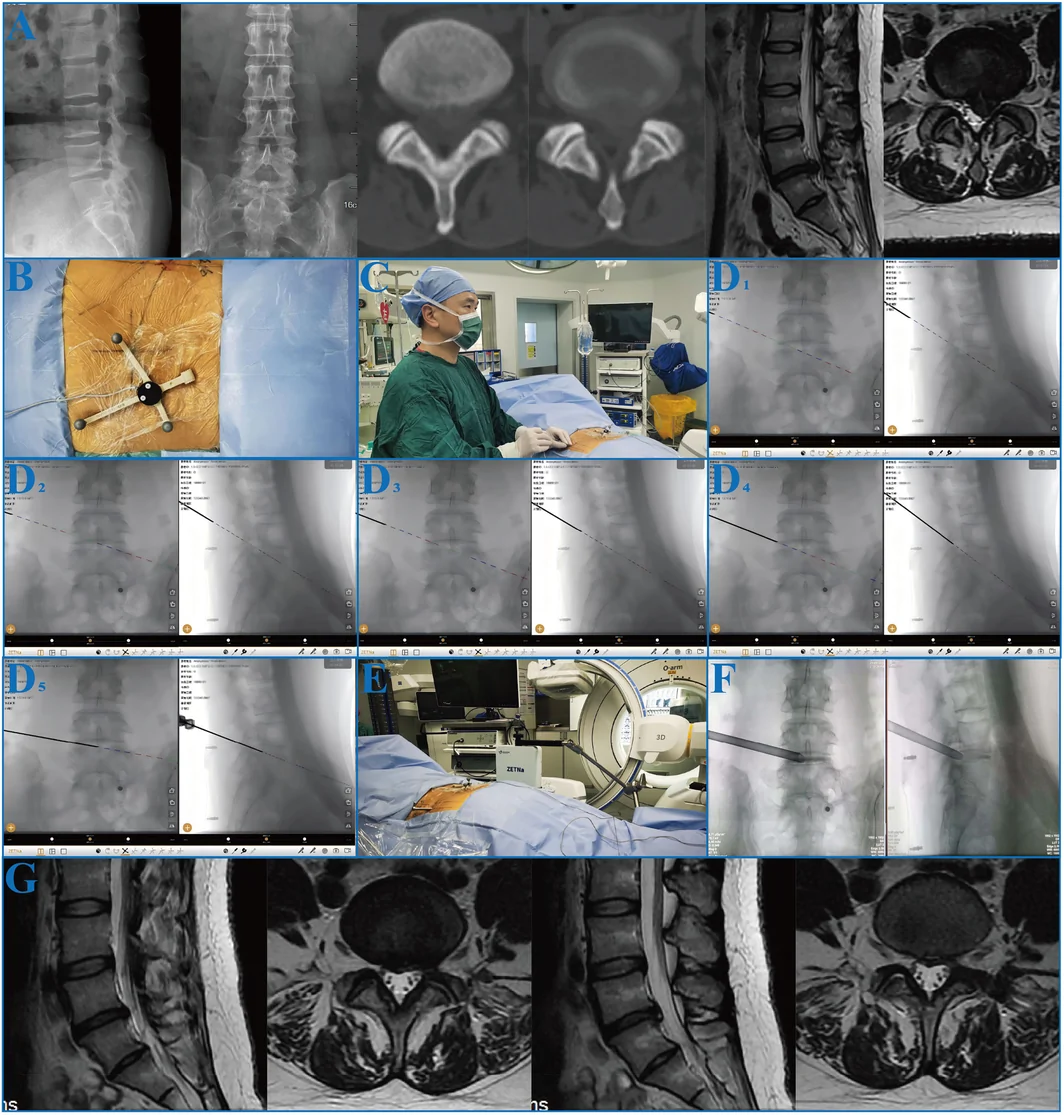
Preoperative, intraoperative, and postoperative imaging of a representative case. (A) Preoperative X‐ray images revealed that the anterior superior iliac spine was aligned with the spinous process of L5. CT imaging revealed significant left posterior protrusion of the L5–S1 intervertebral disc without calcification. MRI revealed a prominent left posterior protrusion of the L5–S1 disc (MSU III). (B) Intraoperative fixation of the reference frame. (C) The surgeon positioned on the left side of the patient, performing the procedure under navigation guidance. (D1–D5) Real‐time navigation displayed the needle's progression through the skin, deep fascia, Zygapophyseal joint, and posterior longitudinal ligament. (E) Establishment of the working channel. (F) After methylene blue injection, fluoroscopy confirmed the accurate placement of the cannula and visualization of the disc. (G) Three‐month postoperative follow‐up MRI showed significant reduction in the L5‐S1 disc herniation and marked alleviation of nerve root compression.

Upon admission, the patient underwent PTED assisted by 2D navigation, and the procedure was successfully completed without complications. Postoperatively, the patient experienced significant relief of back and leg pain and was discharged on the third postoperative day. Follow‐up MRI at 3 months revealed marked reduction of the L5–S1 disc herniation and significant alleviation of nerve root compression (Figure 4G).

## 5 Discussion

### 5.1 Main Clinical Outcomes and Safety

In the present study, we compared 2D electromagnetic navigation‐assisted PTED with 3D electromagnetic navigation‐assisted PTED for the treatment of LDH accompanied by lateral recess stenosis and/or foraminal stenosis. The principal finding was that the 2D workflow was associated with greater procedural efficiency than the 3D workflow, as reflected by shorter navigation registration time, shorter overall operative time, lower radiation exposure, and a shorter learning curve for both the surgeon and the radiologic technologist. Importantly, these workflow‐related advantages were not associated with compromised clinical effectiveness or safety, as both groups achieved substantial postoperative improvement and similarly low complication rates. Although longitudinal analysis identified different temporal recovery patterns for low back pain and ODI between the two groups, favorable final clinical outcomes were obtained in both groups. Taken together, these findings suggest that simplified 2D electromagnetic navigation may represent a more practical and scalable workflow for PTED while preserving the clinical effectiveness of navigation‐assisted surgery.

### 5.2 Procedural Efficiency of 2D Electromagnetic Navigation

PTED was initially employed for the management of LDH [25]. Nevertheless, this technique remains technically demanding and is associated with a substantial learning curve [26]. During the early phase of training, novice surgeons frequently require multiple attempts to accurately reach the target site. Previous studies have suggested that approximately 20 to 31 procedures are necessary for surgeons to significantly reduce puncture and positioning time [26, 27, 28]. With advances in digital navigation technology, increasing attention has been directed toward the use of navigation systems to facilitate PTED, shorten operative time, and improve procedural efficiency [29]. However, many of these studies do not consistently account for the time required for preoperative 3D reconstruction and instrument registration when reporting overall surgical duration [14]. In actual clinical practice, once the time required for 3D image reconstruction and registration is taken into consideration, the total operative time may exceed that of conventional PTED performed by an experienced minimally invasive spine surgeon. In an effort to address these limitations of 3D electromagnetic navigation‐assisted PTED, we applied a 2D electromagnetic navigation‐assisted workflow. Because the prolonged registration process associated with 3D imaging may limit improvements in surgical efficiency, the 2D system was designed on the basis of a permanent calibration strategy, which minimized repeated intraoperative calibration and contributed to a more streamlined registration process [30, 31]. This refinement retains the advantages of navigation guidance while improving the overall usability of the system.

The preoperative setup in the 2D workflow required only a single anteroposterior and lateral fluoroscopic acquisition to upload the intraoperative images and enable instrument tracking. Compared with the 3D electromagnetic navigation system, this approach substantially reduced registration time. In the present study, navigation registration time was significantly shorter in the 2D group than in the 3D group (6.37 ± 0.93 min vs. 12.25 ± 2.11 min; *p* < 0.001). Previous studies have reported a registration time of approximately 10 min for 3D intraoperative navigation, which is broadly consistent with our findings [32]. Several factors may account for the difference observed in our study. First, radiologic technologists required more cases to become familiar with the operation of the 3D system than with the 2D system (23 vs. 14 cases). Second, 3D navigation usually involves more complex spatial registration, image reconstruction, and data processing, all of which inevitably prolong system setup. Third, 3D navigation equipment is generally less mobile and more cumbersome within the operating room, which may further reduce efficiency, particularly in a confined operative environment. Therefore, the shorter registration time in the 2D group appears to reflect a workflow simplification of the navigation workflow rather than a purely technical difference between devices.

In addition, significant differences were observed in total operative time, which included both navigation registration time and skin‐to‐skin duration. Total operative time was significantly shorter in the 2D group than in the 3D group (66.64 ± 9.67 min vs. 88.92 ± 15.98 min; *p* < 0.001). In addition to the shorter registration process, this difference may also be related to the fact that the 2D electromagnetic navigation workflow more closely resembles the procedural logic of conventional PTED. This familiarity may facilitate intraoperative adaptation for surgeons who already have experience with fluoroscopy‐guided PTED, thereby reducing disruption to established operative habits. By contrast, although 3D electromagnetic navigation provides richer spatial information, its more complex registration process and distinct workflow may increase cognitive and operational burden during the procedure. In this regard, a smoother workflow may help maintain operative continuity and improve procedural efficiency [33].

### 5.3 Radiological Advantages and Learning Curve of 2D Electromagnetic Navigation

Compared with conventional C‐arm fluoroscopy requiring approximately 20 exposures, both navigation‐assisted techniques substantially reduced the frequency of intraoperative fluoroscopy (2D group: 2 exposures; 3D group: 1 exposure), which is in line with previous reports [34, 35, 36]. Nevertheless, the radiation dose was significantly lower in the 2D group than in the 3D group. This difference is likely attributable to the reduced imaging burden required for the 2D workflow, in which registration could be completed using only one anteroposterior and one lateral fluoroscopic image before puncture was performed under real‐time electromagnetic guidance. In contrast, 3D navigation requires O‐arm fluoroscopy and CT‐based reconstruction, both of which increase imaging acquisition time and radiation exposure. Previous literature has suggested that the radiation dose associated with a single O‐arm scan may be approximately four to five times higher than that of a single anteroposterior and one lateral fluoroscopic acquisition used in 2D navigation [37]. Therefore, the 2D workflow appears to be associated with a potential advantage in reducing radiation exposure while maintaining surgical efficiency and safety, which is particularly relevant in minimally invasive spine surgery.

With respect to the learning curve, the present study showed that the 2D group reached proficiency earlier than the 3D group for both the surgeon (16 vs. 26 cases) and the radiologic technologist (14 vs. 23 cases). This difference may be explained by the fact that the 2D navigation workflow preserves much of the cognitive and procedural logic of conventional PTED. Surgeons already familiar with conventional PTED are therefore likely to encounter fewer barriers when transitioning to the 2D system. By contrast, although 3D electromagnetic navigation offers more comprehensive spatial information, its more complex registration process and distinct operational interface may require more prolonged adaptation. A similar explanation may apply to the radiologic technologist, for whom the 2D system appears operationally less demanding than the 3D system. Taken together, these findings suggest that 2D navigation may lower the threshold for adoption of navigation‐assisted PTED and may facilitate adoption in centers with varying levels of technical experience.

### 5.4 Application and Limitations of 2D Electromagnetic Navigation

Despite these workflow‐related advantages, the simplified 2D electromagnetic navigation system also has inherent technical limitations that should be acknowledged. Unlike 3D navigation, the 2D workflow relies on anteroposterior and lateral fluoroscopic image registration rather than volumetric reconstruction, and therefore provides less comprehensive spatial information during trajectory planning and needle advancement. As a result, its accuracy may depend more heavily on the quality of the initial fluoroscopic images, the stability of the reference frame, and the operator's ability to integrate navigation information with anatomical experience. In addition, electromagnetic tracking may be affected by environmental interference or subtle displacement of the registration setup, which could reduce navigation reliability in technically demanding situations. These characteristics suggest that the 2D system should be regarded as a simplified and efficient navigation workflow rather than a universal substitute for more information‐rich navigation strategies in all clinical scenarios.

In terms of clinical recovery, both groups achieved significant postoperative improvement in pain and functional status over time. Longitudinal analysis using linear mixed‐effects models demonstrated significant time effects for VAS of back, VAS of leg, and ODI, confirming the overall effectiveness of both navigation‐assisted PTED techniques. No significant main effect of group was observed for these outcomes, indicating that neither technique showed a consistent overall advantage in absolute pain or disability scores throughout follow‐up. However, the group‐by‐time interaction was significant for VAS of back and ODI, whereas no significant interaction was identified for VAS of leg. These findings suggest that although the overall degree of postoperative improvement was comparable between groups, the temporal patterns of recovery in low back pain and disability differed during follow‐up. Notably, both groups achieved favorable final clinical outcomes, with no significant differences in modified MacNab ratings or complication rates. Taken together, these results indicate that 2D electromagnetic navigation‐assisted PTED provides clinical improvement comparable to that of 3D navigation‐assisted PTED, while offering advantages in operative efficiency, radiation reduction, and learning burden.

## 6 Limitations

This study has several limitations that should be acknowledged. First, its retrospective, single‐center design and relatively small sample size may have introduced selection bias and limited the generalizability of the findings. Prospective multicenter studies with larger cohorts and longer follow‐up are needed to confirm the reproducibility and external validity of the present results. Second, treatment allocation was not randomized. Because the choice of navigation workflow was made after preoperative discussion with the patient, selection bias and residual confounding cannot be fully excluded, even though the baseline characteristics appeared comparable between groups. Unmeasured factors, such as patient preference, anatomical subtlety not captured in routine variables, or surgeon‐specific decision‐making, may have influenced treatment selection and perioperative outcomes. Third, the study population was limited to patients with relatively standard anatomical characteristics and straightforward surgical indications. According to the inclusion and exclusion criteria, only patients with single‐level LDH and complete follow‐up were included, whereas patients with previous lumbar surgery, spinal instability, or other complex pathological conditions were excluded. Including patients with severe deformity, marked anatomical variation, or complex revision pathology would likely have introduced substantial heterogeneity in operative difficulty and intraoperative decision‐making, thereby confounding the comparison of workflow‐related outcomes. Therefore, the present findings are most directly applicable to routine primary PTED cases and may not be generalizable to patients with severe spinal deformity, marked anatomical variation, or complex revision surgery. Further studies are needed to determine whether the simplified 2D electromagnetic navigation workflow remains sufficiently accurate and efficient in these more technically challenging settings.

## 7 Conclusion

2D electromagnetic navigation‐assisted PTED is a safe and clinically effective alternative to 3D electromagnetic navigation‐assisted PTED for the treatment of LDH. Compared with the 3D workflow, the 2D approach is associated with greater procedural efficiency, reduced radiation exposure, and a shorter learning curve. Although longitudinal analysis revealed different recovery patterns over time for low back pain and ODI between the two groups, both techniques achieved substantial postoperative improvement, favorable final clinical outcomes, and comparable safety. These findings suggest that simplified 2D electromagnetic navigation may represent a more practical and scalable option for routine clinical application.

## Author Contributions

**Zhong Wei:** writing – original draft, methodology, software, investigation, writing – review and editing, validation. **Haoyu Cheng:** methodology, validation, investigation, writing – original draft. **Ren Gao:** writing – original draft, investigation, validation, methodology. **Xuepeng Wang:** investigation, methodology. **Kangheng Niu:** validation, software, investigation. **Zhenzhen Tao:** investigation, methodology. **Hao An:** validation, investigation. **Biwang Huang:** conceptualization, writing – review and editing, project administration, supervision, data curation. **Chengjie Xiong:** conceptualization, writing – review and editing, project administration, supervision, data curation, funding acquisition. **Junhong Lian:** conceptualization, writing – review and editing, project administration, supervision, data curation, formal analysis.

## Funding

This study was funded by the Wuhan Knowledge Innovation Special Project (2023020201010177), the 2024 Hubei Provincial Natural Science Foundation General Program Project (2024AFB1063), and the Hubei Province Health Science and Technology Project (WJ2025M049).

## Ethics Statement

The study adhered to the Declaration of Helsinki and was approved by the Ethics Committee of our institution (Approval No. [2026]027‐01). Written informed consent was obtained from all patients.

## Conflicts of Interest

The authors declare no conflicts of interest.

## Data Availability

The data that support the findings of this study are available from the corresponding author upon reasonable request.

## References

[1] Global, Regional, and National Incidence, Prevalence, and Years Lived With Disability for 328 Diseases and Injuries for 195 Countries, 1990‐2016: A Systematic Analysis for the Global Burden of Disease Study 2016,” Lancet 390, no. 10100 (2017): 1211–1259. 10.1016/S0140-6736(17)32154-2 PMC5605509 28919117

[2] T. Oichi , Y. Taniguchi , Y. Oshima , S. Tanaka , and T. Saito , “Pathomechanism of Intervertebral Disc Degeneration,” JOR Spine 3, no. 1 (2020): e1076. 10.1002/jsp2.1076 PMC7084053 32211588

[3] M. Kahere and T. Ginindza , “Mapping Evidence on the Prevalence, Incidence, Risk Factors and Cost Associated With Chronic Low Back Pain Among Adults in Sub‐Saharan Africa: A Systematic Scoping Review Protocol,” Systematic Reviews 9, no. 1 (2020): 57. 10.1186/s13643-020-01321-w PMC7077148 32183868

[4] Y. Jin , A. Liu , J. R. Overbey , et al., “Risk Factors for Surgical Intervention in Patients With Primary Spinal Infection on Initial Presentation,” Journal of Neurosurgery. Spine 37, no. 2 (2022): 283–291. 10.3171/2021.12.SPINE21811 PMC11580788 35120318

[5] S. Lener , W. Schmölz , A. Abramovic , P. Kluger , C. Thomé , and S. Hartmann , “The Effect of Various Options for Decompression of Degenerated Lumbar Spine Motion Segments on the Range of Motion: A Biomechanical In Vitro Study,” European Spine Journal 32, no. 4 (2023): 1358–1366. 10.1007/s00586-023-07587-7 36826599

[6] S. Kapetanakis , G. Gkasdaris , A. G. Angoules , and P. Givissis , “Transforaminal Percutaneous Endoscopic Discectomy Using Transforaminal Endoscopic Spine System Technique: Pitfalls That a Beginner Should Avoid,” World Journal of Orthopedics 8, no. 12 (2017): 874–880. 10.5312/wjo.v8.i12.874 PMC5745429 29312845

[7] G. Xu , X. Zhang , M. Zhu , et al., “Clinical Efficacy of Transforaminal Endoscopic Discectomy in the Treatment of Recurrent Lumbar Disc Herniation: A Single‐Center Retrospective Analysis,” BMC Musculoskeletal Disorders 24, no. 1 (2023): 24. 10.1186/s12891-023-06148-9 PMC9835219 36631884

[8] I. Tornatore , A. Basile , A. Aureli , A. Tarantino , G. Orlando , and R. Buharaja , “Effectiveness and Safety of Transforaminal Spinal Endoscopy: Analysis of 1000 Clinical Cases,” Diagnostics (Basel) 15, no. 8 (2025): 1021. 10.3390/diagnostics15081021 PMC12025548 40310433

[9] S. Liu , X. Zhang , Y. Xiong , and H. He , “Minimally Invasive Surgery for Lumbar Disc Herniation: A Meta‐Analysis of Efficacy and Safety,” International Journal of Surgery 111, no. 8 (2025): 5623–5636. 10.1097/JS9.0000000000002434 40434733

[10] P. S. Gadjradj , M. W. van Tulder , C. M. Dirven , W. C. Peul , and B. S. Harhangi , “Clinical Outcomes After Percutaneous Transforaminal Endoscopic Discectomy for Lumbar Disc Herniation: A Prospective Case Series,” Neurosurgical Focus 40, no. 2 (2016): E3. 10.3171/2015.10.FOCUS15484 26828884

[11] P. S. Gadjradj , S. M. Rubinstein , W. C. Peul , et al., “Full Endoscopic Versus Open Discectomy for Sciatica: Randomised Controlled Non‐Inferiority Trial,” BMJ 376 (2022): e065846. 10.1136/bmj-2021-065846 PMC8859734 35190388

[12] H. Wang , B. Huang , C. Li , et al., “Learning Curve for Percutaneous Endoscopic Lumbar Discectomy Depending on the Surgeon's Training Level of Minimally Invasive Spine Surgery,” Clinical Neurology and Neurosurgery 115, no. 10 (2013): 1987–1991. 10.1016/j.clineuro.2013.06.008 23830496

[13] B. Wang , G. Lü , A. A. Patel , P. Ren , and I. Cheng , “An Evaluation of the Learning Curve for a Complex Surgical Technique: The Full Endoscopic Interlaminar Approach for Lumbar Disc Herniations,” Spine Journal 11, no. 2 (2011): 122–130. 10.1016/j.spinee.2010.12.006 21296295

[14] B. Wu , T. Wei , Z. Yao , et al., “A Real‐Time 3D Electromagnetic Navigation System for Percutaneous Transforaminal Endoscopic Discectomy in Patients With Lumbar Disc Herniation: A Retrospective Study,” BMC Musculoskeletal Disorders 23, no. 1 (2022): 57. 10.1186/s12891-022-05012-6 PMC8764808 35039040

[15] G. Fan , R. Han , X. Gu , et al., “Navigation Improves the Learning Curve of Transforamimal Percutaneous Endoscopic Lumbar Discectomy,” International Orthopaedics 41, no. 2 (2017): 323–332. 10.1007/s00264-016-3281-5 27591770

[16] A. K. Sharma , R. G. de Oliveira , S. Suvithayasiri , et al., “The Utilization of Navigation and Emerging Technologies With Endoscopic Spine Surgery: A Narrative Review,” Neurospine 22, no. 1 (2025): 105–117. 10.14245/ns.2449404.702 PMC12010863 40211520

[17] S. C. Overley , S. K. Cho , A. I. Mehta , and P. M. Arnold , “Navigation and Robotics in Spinal Surgery: Where Are we Now?,” Neurosurgery 80, no. 3s (2017): S86–s99. 10.1093/neuros/nyw077 28350944

[18] B. C. Meyer , O. Peter , M. Nagel , et al., “Electromagnetic Field‐Based Navigation for Percutaneous Punctures on C‐Arm CT: Experimental Evaluation and Clinical Application,” European Radiology 18, no. 12 (2008): 2855–2864. 10.1007/s00330-008-1063-1 18545999

[19] D. R. Xu , L. R. Luan , X. X. Ma , Z. C. Cong , and C. L. Zhou , “Comparison of Electromagnetic and Optical Navigation Assisted Endo‐TLIF in the Treatment of Lumbar Spondylolisthesis,” BMC Musculoskeletal Disorders 23, no. 1 (2022): 522. 10.1186/s12891-022-05443-1 PMC9158260 35650587

[20] E. C. Huskisson , “Measurement of Pain,” Lancet 2, no. 7889 (1974): 1127–1131. 10.1016/s0140-6736(74)90884-8 4139420

[21] J. C. T. Fairbank , J. B. Davies , J. Couper , and J. P. O'Brien , “The Oswestry Low Back Pain Disability Questionnaire,” Physiotherapy 66, no. 8 (1980): 271–273. 6450426

[22] I. Macnab , “Negative Disc Exploration. An Analysis of the Causes of Nerve‐Root Involvement in Sixty‐Eight Patients,” Journal of Bone and Joint Surgery (American Volume) 53, no. 5 (1971): 891–903. 4326746

[23] Z. Li , S. Wu , X. Tang , et al., “Evaluating the Learning Curve for Flexible Ureteroscopic Thulium Fiber Laser Lithotripsy Using CUSUM Analysis,” International Journal of Surgery 111, no. 3 (2025): 2430–2438. 10.1097/JS9.0000000000002246 PMC12372719 39903570

[24] J. Xu , D. Wang , J. Liu , et al., “Learning Curve and Complications of Unilateral Biportal Endoscopy: Cumulative Sum and Risk‐Adjusted Cumulative Sum Analysis,” Neurospine 19, no. 3 (2022): 792–804. 10.14245/ns.2143116.558 PMC9537833 35996762

[25] K. U. Lewandrowski , “Incidence, Management, and Cost of Complications After Transforaminal Endoscopic Decompression Surgery for Lumbar Foraminal and Lateral Recess Stenosis: A Value Proposition for Outpatient Ambulatory Surgery,” International Journal of Spine Surgery 13, no. 1 (2019): 53–67. 10.14444/6008 PMC6383464 30805287

[26] V. J. Fleiderman , B. J. Lecaros , T. J. I. Cirillo , F. Álvarez Lemos , V. P. Osorio , and B. N. Wolff , “Transforaminal Endoscopic Lumbar Discectomy: Learning Curve of a Single Surgeon,” Journal of Spine Surgery 9, no. 2 (2023): 159–165. 10.21037/jss-22-54 PMC10331491 37435320

[27] S. Son , Y. Ahn , S. G. Lee , et al., “Learning Curve of Percutaneous Endoscopic Transforaminal Lumbar Discectomy by a Single Surgeon,” Medicine (Baltimore) 100, no. 4 (2021): e24346. 10.1097/MD.0000000000024346 PMC7850775 33530228

[28] O. Maayan , A. Pajak , P. Shahi , et al., “Percutaneous Transforaminal Endoscopic Discectomy Learning Curve: A CuSum Analysis,” Spine (Phila Pa 1976) 48, no. 21 (2023): 1508–1516. 10.1097/BRS.0000000000004730 37235810

[29] X. C. Yao , J. P. Liu , X. R. Du , et al., “Integrated Optical and Magnetic Navigation for Simplified Percutaneous Transforaminal Endoscopic Lumbar Discectomy: A Novel Approach,” Neurospine 22, no. 1 (2025): 297–307. 10.14245/ns.2448750.375 PMC12010866 39842399

[30] W. Xia , Z. Wu , R. Zuo , et al., “Feasibility and Accuracy Evaluation of Novel 2D Instant Navigation System on Spinal Surgery ‐ a Preclinical Study,” BMC Musculoskeletal Disorders 26, no. 1 (2025): 510. 10.1186/s12891-025-08769-8 PMC12100916 40410739

[31] Z. Wu , Y. Shu , J. Ling , et al., “PerCal: A Novel Permanent Calibration Method for Spinal Surgery Navigation,” IEEE Transactions on Instrumentation and Measurement 73 (2024): 1–15.

[32] E. Tabaraee , A. G. Gibson , D. G. Karahalios , E. A. Potts , J. P. Mobasser , and S. Burch , “Intraoperative Cone Beam‐Computed Tomography With Navigation (O‐ARM) Versus Conventional Fluoroscopy (C‐ARM): A Cadaveric Study Comparing Accuracy, Efficiency, and Safety for Spinal Instrumentation,” Spine (Phila Pa 1976) 38, no. 22 (2013): 1953–1958. 10.1097/BRS.0b013e3182a51d1e 23883830

[33] S. A. McQueen , M. Hammond , A. L. McParland , R. R. Sonnadara , and C. A. Moulton , “Reigniting the Flame: An Exploration of Flow in Surgery,” Academic Medicine 100, no. 3 (2025): 313–318. 10.1097/ACM.0000000000005920 40009722

[34] Y. Guo , H. Pei , Y. Dong , Q. Sun , K. Kang , and H. Gao , “C‐Arm‐Guided Versus Ultrasound Volumetric Navigation‐Guided Percutaneous Transforaminal Endoscopic Discectomy for the Treatment of Lumbar Disc Herniation: A Retrospective Study,” Medicine (Baltimore) 103, no. 41 (2024): e39962. 10.1097/MD.0000000000039962 PMC11479475 39465857

[35] J. Gong , X. Huang , L. Luo , et al., “Radiation Dose Reduction and Surgical Efficiency Improvement in Endoscopic Transforaminal Lumbar Interbody Fusion Assisted by Intraoperative O‐Arm Navigation: A Retrospective Observational Study,” Neurospine 19, no. 2 (2022): 376–384. 10.14245/ns.2143324.662 PMC9260536 35793934

[36] W. Guo , G. Xia , X. Dong , X. Zhang , W. Zhang , and B. Liao , “Comparative Analysis of Operative Time, Blood Loss, and X‐Ray Parameters: Two‐Dimensional Navigation Guided Percutaneous Endoscopic Transforaminal Discectomy vs. Conventional Microscopic Surgery for Lumbar Disc Herniation,” Journal of Radiation Research and Applied Science 17, no. 4 (2024): 101142.

[37] M. Boudissa , P. Girard , G. Kerschbaumer , S. Ruatti , and J. Tonetti , “Does Intraoperative 3D Navigation Improve SpineJack Vertebral Augmentation in Thoracic and Lumbar Compression Fracture?,” Orthopaedics & Traumatology, Surgery & Research 107, no. 7 (2021): 103028. 10.1016/j.otsr.2021.103028 34329760

